# Resonant Mode Reduction in Radiofrequency Volume Coils for Ultrahigh Field Magnetic Resonance Imaging

**DOI:** 10.3390/ma4081333

**Published:** 2011-07-28

**Authors:** Yong Pang, Zhentian Xie, Ye Li, Duan Xu, Daniel Vigneron, Xiaoliang Zhang

**Affiliations:** 1 Department of Radiology and Biomedical Imaging, University of California San Francisco, San Francisco, CA 94158, USA; E-Mails: yong.pang@ucsf.edu (Y.P.); xiezht3r@gmail.com (Z.X.); ye.li@ucsf.edu (Y.L.); duan.xu@ucsf.edu (D.X.); dan.vigneron@ucsf.edu (D.V.); 2 UCSF/UC Berkeley Joint Graduate Group in Bioengineering, San Francisco & Berkeley, CA 94720, USA; 3 California Institute for Quantitative Biosciences (QB3), San Francisco, CA 94158, USA

**Keywords:** Finite-Difference Time-Domain, common-mode, microstrip, Magnetic Resonance Imaging, high field

## Abstract

In a multimodal volume coil, only one mode can generate homogeneous Radiofrequency (RF) field for Magnetic Resonance Imaging. The existence of other modes may increase the volume coil design difficulties and potentially decreases coil performance. In this study, we introduce common-mode resonator technique to high and ultrahigh field volume coil designs to reduce the resonant mode while maintain the homogeneity of the RF field. To investigate the design method, the common-mode resonator was realized by using a microstrip line which was split along the central to become a pair of parallel transmission lines within which common-mode currents exist. Eight common-mode resonators were placed equidistantly along the circumference of a low loss dielectric cylinder to form a volume coil. Theoretical analysis and comparison between the 16-strut common-mode volume coil and a conventional 16-strut volume coil in terms of RF field homogeneity and efficiency was performed using Finite-Difference Time-Domain (FDTD) method at 298.2 MHz. MR imaging experiments were performed by using a prototype of the common-mode volume coil on a whole body 7 Tesla scanner. FDTD simulation results showed the reduced number of resonant modes of the common-mode volume coil over the conventional volume coil, while the RF field homogeneity of the two type volume coils was kept at the same level. MR imaging of a water phantom and a kiwi fruit showing the feasibility of the proposed method for simplifying the volume coil design is also presented.

## 1. Introduction

In Magnetic Resonance Imaging (MRI), volume coil is a well-established RF coil type which is capable of creating a nearly homogeneous Radiofrequency (RF) field and clinically-acceptable signal-to-noise ratio (SNR) [1]. Although RF coil arrays [2,3,4,5,6,7,8,9,10,11,12,13] have become more and more popular in MRI applications and essential to parallel transmission [14,15,16,17,18] and parallel imaging [19,20,21,22,23,24,25,26] to shorten the imaging time, RF volume coils still play an important role in MR signal excitation and reception for applications using regular MR methodology [27,28,29,30,31,32,33,34,35,36,37,38,39,40] and also parallel imaging [41,42]. Normally, a multimodal volume coil with *N* rungs or struts resonates at *N*/2 intrinsic modes [1,43,44,45] or *N*/2 + 1 modes [46,47]. Among these resonant modes, there is only one mode which generates a transverse homogeneous RF field for MR imaging in a horizontal static magnetic field. The existence of the other modes may overlap with the homogeneous resonant mode, which makes it difficult to differentiate them and to design the coil [48]. Another practical issue is the coupling among the elements. In order to yield homogeneous RF field distribution, sufficient coupling among the resonant elements (rungs or struts) of a multimodal volume coil is necessary. Although the coupling could be improved by reducing the distances between the elements and increasing the number of the coil elements, in some circumstances these methods are not easy to implement. In addition, increasing the number of coil elements in a volume coil could result in a decrease of coil performance, due to the losses in the capacitors and conductors. The number of resonant elements in a volume coil has to be chosen to reach a good compromise between field homogeneity degree and quality factor for the resonators. 

In this work, we present a novel volume coil design method using common-mode resonator technique to reduce the resonant modes without sacrificing the RF field homogeneity. The common-mode element was realized using a split microstrip resonator that has a pair of parallel transmission lines to support common-mode currents [49]. A 7 Tesla volume coil was built based on this common-mode resonator structure to investigate the feasibility of the proposed volume coil design for mode reduction. Theoretical analysis and comparison between the proposed design and a traditional 16-strut volume coil using Finite-Difference Time-Domain (FDTD) method [50,51,52] was presented. MR imaging of a water phantom and a kiwi fruit was performed using the prototype volume coil on a 7T scanner to validate the common-mode volume coil design method.

## 2. Materials and Methods

**Figure 1:**
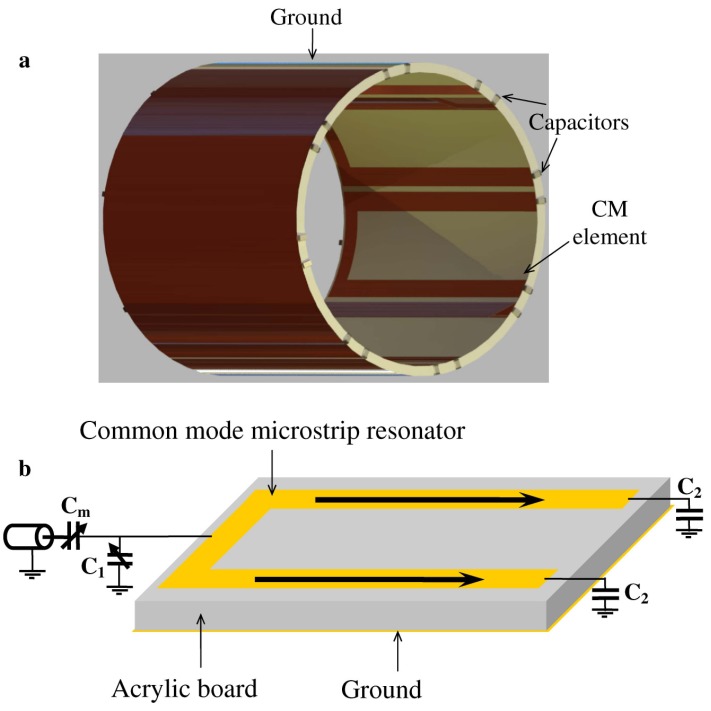
(**a**) Diagram of the 16-strut common-mode microstrip volume coil; (**b**) Diagram of the common-mode microstrip surface coil, where the solid line denotes the common-mode current in the microstrip coil.

The model of the volume coil with 8 common-mode elements (which has 16 struts) is shown in Figure 1a, and the structure of each element is shown in Figure 1b. Each element was modeled as a 3.5” long and 1.25” wide split microstrip resonator [53,54,55,56]. Each strip conductor was split along the central line to form a pair of parallel transmission lines, so that common-mode currents with identical amplitude and phase can be generated within the two legs. The substrate of the microstrip resonator was modeled as a commonly used acrylic board with permittivity of 2.62, while the strip conductor and ground plane were modeled as a thin copper foil. The microstrip resonator were terminated using capacitors (*i.e.*, C_1_ and C_2_ in Figure 1) at both ends. This split microstrip resonator can also be treated as a second harmonic microstrip resonator which supports full sine wave [57]. The resonant frequency of the common-mode element can be estimated by using the following analytic equation:

(1) $$f_{re}=\frac{{\left(2\pi f_{re}Z_{0}\right)}^{2}C_{1}C_{2}-1}{2\pi Z_{0}(C_{1}+C_{2})}\tan(\frac{2\pi l\sqrt{\varepsilon_{eff}}}{c}f_{re})$$

where *C*_1_ and *C*_2_ are the capacitance of the termination capacitors on the microstrip resonator shown in Figure 1. *l* and *ε_eff_* are the length and effective permittivity of the microstrip resonator. *Z*_0_ is the characteristic impedance of the microstrip resonator. In practice, the frequency tuning can be performed by adjusting *C*_1 _and *C*_2_. Then, the resonant frequency *f_vc_* of the common-mode volume coil can be calculated by:

(2) $$f_{vc}=\frac{1}{\sqrt{1+\frac{1}{L}\sum_{i=1}^{N-1}k_{1,i+1}\cos(\frac{2\pi }{N}i)}}f_{re}$$

where *k*_1, i+1 _ is the mutual inductance between the 1st element and the (I + 1)th elements; *f_re_* denotes the resonant frequency of the element; N is the number of element of the volume coil. The capacitances of *C*_1 _and *C*_2 _were 14 pF and 15 pF, respectively, when the volume coil was tuned to 298.2 MHz, while the frequency of each element *f_re_* was 327.1 MHz.

**Figure 2:**
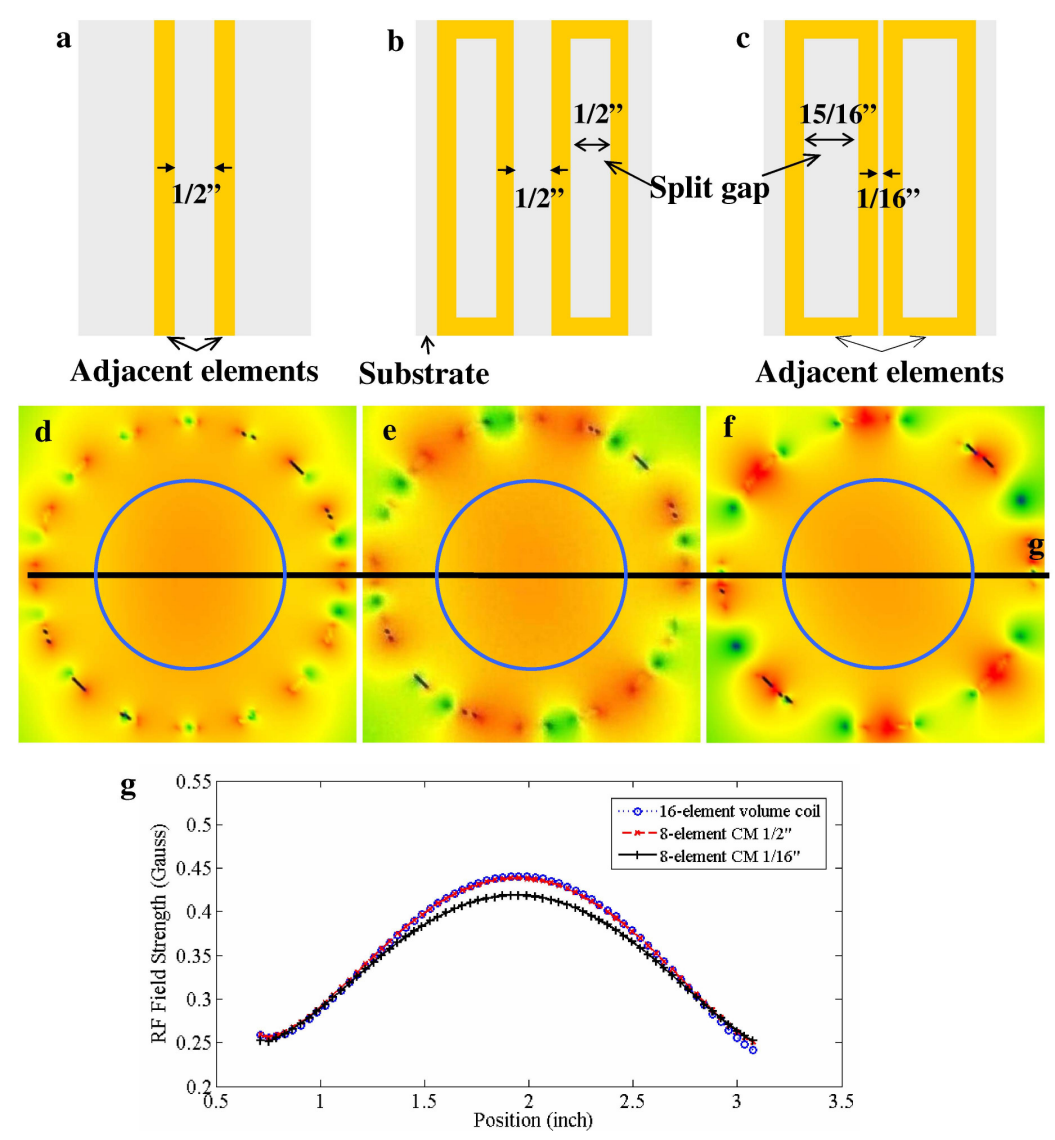
(**a**), (**b**), (**c**) structures of adjacent elements of the typical 16-strut volume coil and two 16-strut common-mode (CM) volume coils with neighboring distance of 1/2 inch and 1/16 inch; (**d**), (**e**), (**f**) the Finite-Difference Time-Domain (FDTD) simulation results of three corresponding models at the central transverse plane; (**g**) 1D profiles of the three magnetic field distribution of the central line. The blue circles in the 2D images denote the area of the 2.4” diameter phantom with permittivity of 58 and conductivity of 0.8 S/m which are close to those of average mouse muscle. The constructive interference of *B_1_*+ pattern on the image is clearly observed. The RF field homogeneity of the CM volume coil is similar to or better than that of a conventional volume coil while the leg number of the CM coil is reduce by half, leading to reduced number of resonant modes. The reduced number of resonant modes helps to simplify the coil design and potentially gain coil efficiency.

For evaluation and comparison of the RF field homogeneity and efficiency, two 16-strut common-mode volume coil models and one typical 16-strut volume coil model were built. The bore sizes of them were the same: 4” OD, 3.75” ID and 4” in length. In the typical 16-strut volume coil the elements were 1/4” in width and uniformly placed on the inner side of the cylinder as shown in Figure 2a. For the two 16-strut common-mode volume coil models the distance between the two neighboring elements were 1/2” and 1/16”, respectively, as shown in Figure 2b and c. In the case of the model with 1/2” distance, the split gap between two legs of one element equaled the distance between the neighboring elements, therefore the 16 legs were uniformly distributed. For the other model with 1/16” distance, the split gap was wider and the neighboring elements were closer to each other. A cylinder phantom with permittivity of 58 and conductivity of 0.8 S/m at 298.2 MHz, which were close to those parameters of average mouse muscle, was modeled within the three coil models. The diameter of the phantom was 2.4” which was about 63% of the coil ID. A series voltage source with 50 Ohm impedance was modeled as the feed-port for each model. The Yee cell size for FDTD mesh was 1 mm in transverse plane and 3 mm in longitudinal direction, which was small enough for satisfying the simulation accuracy and required reasonable computational time. The computational grid size was 138 × 137 × 68. The boundary condition was set as Perfectly Matching Layers (PML) for all boundaries. The stop criteria were that the calculation converged to −35 dB or reached the maximum iteration number of 10^6^. All calculations were performed on the platform of XFDTD 6.4 (Remcom, Inc., State College). 

To evaluate the coil efficiency and RF field homogeneity, the mean and standard deviation were utilized:

(3) $$Mean_{B_{1}}=\frac{\sum_{x\in Phantom}\left|B_{1,x}\right|}{N}$$

(4) $$Std_{B_{1}}=\sqrt{\frac{\sum_{x\in Phantom}{\left(\left|B_{1,x}\right|-Mean_{B_{1}}\right)}^{2}}{N-1}}$$

where *B_1,x_* denotes the RF field strength of the pixel *x* within the phantom area. In the simulation, the input net power for each coil model was scaled to 1 Watt, therefore the average RF strength demonstrates the coil efficiency—how much field strength a coil can generate per unit power. The stronger the average RF field is, the more efficient the coil is. The standard deviation demonstrates the RF field homogeneity—the total variation of the RF field strength within the interested area. The smaller the *Std_B1_* is, the more homogeneous the RF field is.

Finally, bench test and MR tests of the common-mode volume coil with neighboring element distance of 1/16” was performed. By adjusting the capacitance the coil was tuned at 298.2 MHz for ^1^H imaging at 7T. The coil was fed at two quadrature ports separated by 90°. Bench tests on the resonant modes and isolation between the two quadrature ports were measured on the same network analyzer Agilent E5070B. The termination capacitance measurement was conducted on a RCL meter (Fluke PM6303A). In the MR test, a cylindrical water phantom and a kiwi fruit were imaged using the GE 7T whole body MR scanner. A set of fast spin echo images in both the sagittal and axial planes were acquired with TR = 2 s, FA = 45°, 10 mm in plane and 3 mm thick slice, Number of excitation = 1.

## 3. Results

The Gauss wave was used to determine proper capacitances for three volume coil models working at 298.2 MHz and then the Sinusoidal wave was applied to simulate their RF field distributions. The structures and the FDTD simulation results of the typical 16-strut volume coil and the two 16-strut common-mode volume coil models with different neighboring element distance are shown in Figure 2. Due to the use of the average mouse muscle model with cylinder geometry at ultrahigh field of 7T, the constructive interference of *B_1_*+ pattern is clearly observed from the results. The input net power for each coil model was scaled to 1 Watt. From the 2D figures and 1D profiles it is shown that the RF field homogeneity and strength of the three models are almost the same. By using the statistical analysis described in Equation (3) and (4), the average strength and standard deviation of the RF fields within the phantom area of the three different coil models can be evaluated: For the typical 16-element volume coil, 1/2” neighboring element distance and 1/16” distance common-mode volume coils, the average field strengths are 0.36, 0.36 and 0. 34 Gauss, respectively, and the standard deviations are 0.051, 0.051 and 0.049 Gauss, respectively. The normalized standard deviations per Gauss (*i.e*., standard deviation/average field strength) are all about 0.144 Gauss. The 1/2” distance common-mode coil had the same performance as the typical volume coil in both coil efficiency and RF field homogeneity. The 1/16” distance common-mode coil’s efficiency was slightly worse than the 1/2” coil because of the longer length along XY, however the former’s RF field homogeneity was a little better than the latter’s. The advantage of the common-mode volume coil is with the same leg number as a typical volume coil, the resonant mode number can be reduced by half almost without deteriorating the RF field homogeneity or decreasing the coil efficiency. Moreover, the closer distance between adjacent elements is potential to increase their mutual coupling which is helpful for volume coils. Furthermore, the comparison results of the two common-mode volume coil with different split gap also demonstrated the robustness of the proposed method: The RF homogeneity and efficiency were almost not varying with the split gap of the element, which makes the common-mode volume coil design easier and more tolerant of mechanical errors. 

**Figure 3:**
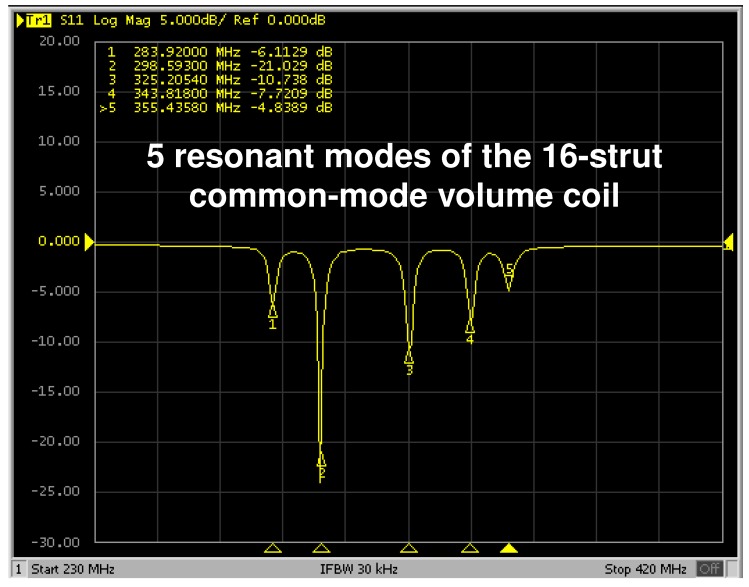
Bench measurement result of reflection coefficient S11 plot of the 16-strut common-mode volume coil. 5 well defined resonant modes can be clearly observed from the picture showing reduction in resonant modes, while a conventional 16-strut volume coil normally has 9 resonant modes (or 8 modes for regular birdcage coil).

The prototype of the 16-strut common-mode microstrip volume coil with 1/16” neighboring distance was built and tuned to 298.2 MHz for bench test and MR test. Well-defined five resonance peaks for proton were clearly identified on the network analyzer, indicating that all the elements were sufficiently coupled as shown in Figure 3. Compared with a conventional 16-strut volume coil which normally has 9 resonant modes (or 8 modes for regular birdcage coil), the resonant mode has been significantly reduced. The transmission coefficient *S*_21_ between two quadrature driving ports was better than −20 dB, showing that the driving ports have been decoupled sufficiently.

**Figure 4:**
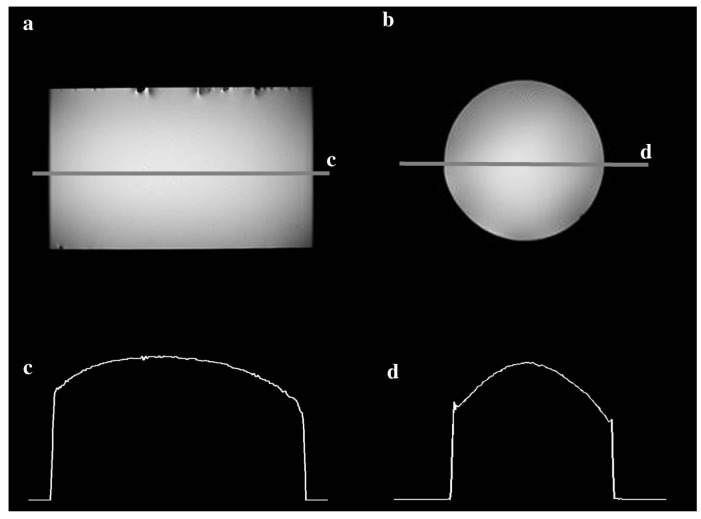
Fast spin echo proton images of a cylindrical water phantom in (**a**) sagittal plane and (**b**) axial plane using the 16-strut common-mode volume coil at 7T; (**c**) and (**d**) are 1D profiles on the central lines of the sagittal image and axial image, respectively. Despite the dielectric resonance effect at the ultrahigh field of 7T, comparatively homogeneous images were obtained using the common-mode volume coil with only 8 elements.

**Figure 5:**
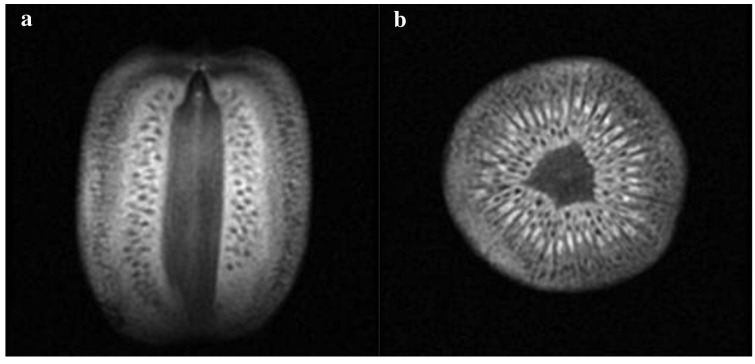
Proton fast spin echo images of a kiwifruit on both (**a**) sagittal plane and (**b**) axial plane acquired using the 16-strut common-mode volume coil at 7T. This imaging result demonstrates the feasibility of the common-mode method for designing volume coils with a lower number of elements but uniform at high fields.

Two fast spin echo images in both sagittal and axial planes from a water phantom with the common-mode microstrip volume coil are shown in Figure 4. Although at ultrahigh field of 298.2 MHz the dielectric resonance effect was clearly observed, the images acquired with the common-mode volume coil were still comparatively homogeneous. By using Equations (3) and (4), the average strength and homogeneity of the RF field on the axial image were 174.6 and 30.4, respectively. The standard deviation was about 17.4% of the average value. Figure 5 shows kiwi fruit images in both sagittal and axial planes acquired from the common-mode volume coil, demonstrating the feasibility of the common-mode method for designing volume coils with a lower number of elements but uniform at high fields.

## 4. Discussion and Conclusions

It has been demonstrated that the common-mode volume coil proposed in this work is able to reduce the resonant modes, simplifying the complicated volume coil design without deteriorating the RF field homogeneity and coil efficiency. Although a common-mode resonator has two struts, such a resonator in a volume coil is considered a resonant element. Therefore, with the same number of struts, the common-mode volume coil has only half the number of resonant elements of a typical volume coil and thus a significantly reduced number of resonant modes. This design method provides an easy-to-implement and efficient approach to volume coil design for generating homogeneous MR images, particularly at high and ultrahigh fields. Furthermore, the common-mode volume coil design is an error-tolerant design: Changing the split gap of the elements generally does not affect the RF coil performance. This makes the common-mode volume coil design easier and more tolerant to mechanical errors. Although microstrip transmission line approach is used in this work to investigate the feasibility of the proposed resonant mode reduction method using common-mode design, practically other coil design techniques, e.g., lumped element methods, can be also utilized to realize this resonant mode reduction technique. 

## References

[1] Hayes C.E., Edelstein W.A., Schenck J.F., Mueller O.M., Eash M. (1985). An efficient, highly homogeneous radiofrequency coil for whole-body NMR imaging at 1.5 T. J. Magn. Resonance.

[2] Roemer P.B., Edelstein W.A., Hayes C.E., Souza S.P., Mueller O.M. (1990). The NMR phased array. Magn. Reson. Med..

[3] Chang G., Friedrich K.M., Wang L., Vieira R.L., Schweitzer M.E., Recht M.P., Wiggins G.C., Regatte R.R. (2010). MRI of the wrist at 7 tesla using an eight-channel array coil combined with parallel imaging: Preliminary results. J. Magn. Reson. Imaging.

[4] Balu N., Yarnykh V.L., Scholnick J., Chu B., Yuan C., Hayes C. (2009). Improvements in carotid plaque imaging using a new eight-element phased array coil at 3T. J. Magn. Reson. Imaging.

[5] Hardy C.J., Giaquinto R.O., Piel J.E., Rohling K.W., Marinelli L., Blezek D.J., Fiveland E.W., Darrow R.D., Foo T.K. (2008). 128-channel body MRI with a flexible high-density receiver-coil array. J. Magn. Reson. Imaging.

[6] Buehrer M., Pruessmann K.P., Boesiger P., Kozerke S. (2007). Array compression for MRI with large coil arrays. Magn. Reson. Med..

[7] Wiggins G.C., Triantafyllou C., Potthast A., Reykowski A., Nittka M., Wald L.L. (2006). 32-channel 3 Tesla receive-only phased-array head coil with soccer-ball element geometry. Magn. Reson. Med..

[8] Hardy C.J., Cline H.E., Giaquinto R.O., Niendorf T., Grant A.K., Sodickson D.K. (2006). 32-element receiver-coil array for cardiac imaging. Magn. Reson. Med..

[9] Zhu Y., Hardy C.J., Sodickson D.K., Giaquinto R.O., Dumoulin C.L., Kenwood G., Niendorf T., Lejay H., McKenzie C.A., Ohliger M.A. (2004). Highly parallel volumetric imaging with a 32-element RF coil array. Magn. Reson. Med..

[10] Henry R.G., Fischbein N.J., Dillon W.P., Vigneron D.B., Nelson S.J. (2001). High-sensitivity coil array for head and neck imaging: Technical note. AJNR Am. J. Neuroradiol..

[11] Weiger M., Pruessmann K.P., Leussler C., Roschmann P., Boesiger P. (2001). Specific coil design for SENSE: A six-element cardiac array. Magn. Reson. Med..

[12] Porter J.R., Wright S.M., Reykowski A. (1998). A 16-element phased-array head coil. Magn. Reson. Med..

[13] Hayes C.E., Tsuruda J.S., Mathis C.M. (1993). Temporal lobes: Surface MR coil phased-array imaging. Radiology.

[14] Zhu Y. (2004). Parallel excitation with an array of transmit coils. Magn. Reson. Med..

[15] Katscher U., Bornert P., Leussler C., van den Brink J.S. (2003). Transmit SENSE. Magn. Reson. Med..

[16] Grissom W., Yip C.Y., Zhang Z., Stenger V.A., Fessler J.A., Noll D.C. (2006). Spatial domain method for the design of RF pulses in multicoil parallel excitation. Magn. Reson. Med..

[17] Ma C., Xu D., King K.F., Liang Z.P. (2011). Joint design of spoke trajectories and RF pulses for parallel excitation. Magn. Reson. Med..

[18] Xu D., King K.F., Zhu Y., McKinnon G.C., Liang Z.P. (2007). A noniterative method to design large-tip-angle multidimensional spatially-selective radio frequency pulses for parallel transmission. Magn. Reson. Med..

[19] Sodickson D.K., Manning W.J. (1997). Simultaneous acquisition of spatial harmonics (SMASH): Fast imaging with radiofrequency coil arrays. Magn. Reson. Med..

[20] Pruessmann K.P., Weiger M., Scheidegger M.B., Boesiger P. (1999). SENSE: Sensitivity encoding for fast MRI. Magn. Reson. Med..

[21] Griswold M.A., Jakob P.M., Heidemann R.M., Nittka M., Jellus V., Wang J., Kiefer B., Haase A. (2002). Generalized autocalibrating partially parallel acquisitions (GRAPPA). Magn. Reson. Med..

[22] Xu D., King K.F., Liang Z.P. (2007). Improving k-t SENSE by adaptive regularization. Magn. Reson. Med..

[23] Cukur T., Santos J.M., Pauly J.M., Nishimura D.G. (2010). Variable-density parallel imaging with partially localized coil sensitivities. IEEE Trans. Med. Imaging.

[24] Lustig M., Pauly J.M. (2010). SPIRiT: Iterative self-consistent parallel imaging reconstruction from arbitrary k-space. Magn. Reson. Med..

[25] Ji J., Wright S. (2005). Parallel MR imaging with accelerations beyond the number of receiver channels using real image reconstruction. Conf. Proc. IEEE Eng. Med. Biol. Soc..

[26] Li Y., Xie Z., Pang Y., Vigneron D., Zhang X. (2011). ICE decoupling technique for RF coil array designs. Med. Phys..

[27] Vaughan J.T., Adriany G., Snyder C.J., Tian J., Thiel T., Bolinger L., Liu H., DelaBarre L., Ugurbil K. (2004). Efficient high-frequency body coil for high-field MRI. Magn. Reson. Med..

[28] Zhang X., Ugurbil K., Chen W. (2003). A microstrip transmission line volume coil for human head MR imaging at 4T. J. Magn. Reson..

[29] Wiggins G.C., Potthast A., Triantafyllou C., Wiggins C.J., Wald L.L. (2005). Eight-channel phased array coil and detunable TEM volume coil for 7 T brain imaging. Magn. Reson. Med..

[30] Avdievich N.I., Hetherington H.P. (2007). 4 T Actively detuneable double-tuned 1H/31P head volume coil and four-channel 31P phased array for human brain spectroscopy. J. Magn. Reson..

[31] Vaughan J.T., Adriany G., Garwood M., Yacoub E., Duong T., DelaBarre L., Andersen P., Ugurbil K. (2002). Detunable transverse electromagnetic (TEM) volume coil for high-field NMR. Magn. Reson. Med..

[32] Wen H., Chesnick A.S., Balaban R.S. (1994). The design and test of a new volume coil for high field imaging. Magn. Reson. Med..

[33] Kneeland J.B., Jesmanowicz A., Froncisz W., Hyde J.S. (1988). Simultaneous reception from a whole-volume coil and a surface coil on a clinical MR system. Radiology.

[34] Alecci M., Collins C.M., Smith M.B., Jezzard P. (2001). Radio frequency magnetic field mapping of a 3 Tesla birdcage coil: Experimental and theoretical dependence on sample properties. Magn. Reson. Med..

[35] Collins C.M., Smith M.B. (2001). Signal-to-noise ratio and absorbed power as functions of main magnetic field strength, and definition of “90 degrees” RF pulse for the head in the birdcage coil. Magn. Reson. Med..

[36] Fujita H., Braum W.O., Morich M.A. (2000). Novel quadrature birdcage coil for a vertical B(0) field open MRI system. Magn. Reson. Med..

[37] Ibrahim T.S., Lee R., Baertlein B.A., Robitaille P.M. (2001). B1 field homogeneity and SAR calculations for the birdcage coil. Phys. Med. Biol..

[38] Jin J., Shen G., Perkins T. (1994). On the field inhomogeneity of a birdcage coil. Magn. Reson. Med..

[39] Shen G.X., Boada F.E., Thulborn K.R. (1997). Dual-frequency, dual-quadrature, birdcage RF coil design with identical B1 pattern for sodium and proton imaging of the human brain at 1.5 T. Magn. Reson. Med..

[40] Murphy-Boesch J., Srinivasan R., Carvajal L., Brown T.R. (1994). Two configurations of the four-ring birdcage coil for 1H imaging and 1H-decoupled 31P spectroscopy of the human head. J. Magn. Reson. B.

[41] Lin F.H., Kwong K.K., Huang I.J., Belliveau J.W., Wald L.L. (2003). Degenerate mode birdcage volume coil for sensitivity-encoded imaging. Magn. Reson. Med..

[42] Wang C., Qu P., Shen G.X. (2006). Potential advantage of higher-order modes of birdcage coil for parallel imaging. J. Magn. Reson..

[43] Joseph P.M., Lu D. (1989). A technique for double resonant operation of birdcage imaging coils. IEEE Trans. Med. Imaging.

[44] Tropp J. (1989). The theory of the birdcage resonator. J. Magn. Reson. Imaging.

[45] Vullo T., Zipagan R.T., Pascone R., Whalen J.P., Cahill P.T. (1992). Experimental design and fabrication of birdcage resonators for magnetic resonance imaging. Magn. Reson. Med..

[46] Vaughan J.T., Hetherington H.P., Otu J.O., Pan J.W., Pohost G.M. (1994). High frequency volume coils for clinical NMR imaging and spectroscopy. Magn. Reson. Med..

[47] Zhang X., Ugurbil K., Sainati R., Chen W. (2005). An inverted-microstrip resonator for human head proton MR imaging at 7 tesla. IEEE Trans. Biomed. Eng..

[48] Duan Y., Peterson B.S., Liu F., Brown T.R., Ibrahim T.S., Kangarlu A. (2009). Computational and experimental optimization of a double-tuned (1)H/(31)P four-ring birdcage head coil for MRS at 3T. J. Magn. Reson. Imaging.

[49] Xie Z., Xu D., Vigneron D.B., Zhang X. Common mode volume coil design for in vivo MR imaging at 7T. Proceedings of the 17th Annual Meeting of ISMRM.

[50] Sekino M., Kim D., Ohsaki H. (2008). Finite-difference time-domain simulations of radio frequency electromagnetic fields and signal inhomogeneities in ultrahigh-field magnetic resonance imaging systems. J. Appl. Phys..

[51] Collins C.M., Smith M.B. (2001). Calculations of B(1) distribution, SNR, and SAR for a surface coil adjacent to an anatomically-accurate human body model. Magn. Reson. Med..

[52] Yang Q.X., Wang J., Zhang X., Collins C.M., Smith M.B., Liu H., Zhu X.H., Vaughan J.T., Ugurbil K., Chen W. (2002). Analysis of wave behavior in lossy dielectric samples at high field. Magn. Reson. Med..

[53] Lee R.F., Westgate C.R., Weiss R.G., Newman D.C., Bottomley P.A. (2001). Planar strip array (PSA) for MRI. Magn. Reson. Med..

[54] Zhang X., Ugurbil K., Chen W. (2001). Microstrip RF surface coil design for extremely high-field MRI and spectroscopy. Magn. Reson. Med..

[55] Wu B., Wang C., Kelley D.A., Xu D., Vigneron D.B., Nelson S.J., Zhang X. (2010). Shielded microstrip array for 7T human MR imaging. IEEE Trans. Med. Imaging.

[56] Wu B., Wang C., Krug R., Kelley D.A., Xu D., Pang Y., Banerjee S., Vigneron D.B., Nelson S.J., Majumdar S., Zhang X. (2010). 7T human spine imaging arrays with adjustable inductive decoupling. IEEE Trans. Biomed. Eng..

[57] Zhang X., Zhu X.H., Chen W. (2005). Higher-order harmonic transmission-line RF coil design for MR applications. Magn. Reson. Med..

